# Molecular cloning and functional analysis of a plastidial ω3 desaturase from *Emiliania huxleyi*

**DOI:** 10.3389/fmicb.2024.1381097

**Published:** 2024-07-11

**Authors:** Kaiwen Sun, Dauenpen Meesapyodsuk, Xiao Qiu

**Affiliations:** ^1^Department of Food and Bioproduct Sciences, University of Saskatchewan, Saskatoon, SK, Canada; ^2^National Research Council of Canada, Saskatoon, SK, Canada

**Keywords:** polyunsaturated fatty acids, octadecapentaenoic acid, docosahexaenoic acid, plastidial ω3 desaturase, *Synechococcus elongatus*, *Emiliania huxleyi*

## Abstract

*Emiliania huxleyi* is a marine microalga playing a significant ecological and biogeochemical role in oceans. It can produce several polyunsaturated fatty acids (PUFAs), such as docosahexaenoic acid (DHA, 22:6–4,7,10,13,16,19) and octadecapentaenoic acid (OPA, 18:5–3,6,9,12,15), providing a primary source for nutritionally important ω3 PUFAs in the marine food chain. However, the biosynthesis of these PUFAs in this organism is not well understood. In this study, a full length plastidial ω3 desaturase cDNA (*EhN3*) was cloned from this alga. Heterologous expression of EhN3 with and without the chloroplast targeting peptide (cTP) in cyanobacterium *Synechococcus elongatus* showed that it possessed high desaturation activity toward C18-ω6 PUFAs, linoleic acid (LA, 18:2–9,12), γ-linolenic acid (GLA, 18:3–6,9,12), and C20-ω6 PUFAs, dihomo-γ-linolenic acid (DGLA, 20:3–8,11,14) and arachidonic acid (ARA, 20:4–5,8,11,14) that were exogenously supplied. Desaturation efficiency could reach almost 100% in a time course. On the other hand, when expressed in *Saccharomyces cerevisiae*, EhN3 with and without cTP did not exhibit any activity. Lipid analysis of *Synechococcus* transformants expressing EhN3 showed that it utilized galactolipids as substrates. Transcriptional expression analysis revealed that the expression of the gene increased while the growth temperature decreased, which was correlated with the increased production of ω3-PUFAs, particularly OPA. This is the first report of a plastidial ω3 desaturase from microalgae that can effectively introduce an ω3 double bond into both C18-ω6 and C20-ω6 PUFAs. EhN3 might also be one of the key enzymes involved in the biosynthesis of OPA in *E. huxleyi* through the plastidial aerobic pathway.

## Introduction

Omega-3 polyunsaturated fatty acids (PUFAs) are a group of PUFAs that have the last double bond at the third carbon position away from the methyl end and are vital for human health and wellbeing. Among ω3 PUFAs, α-linolenic acid (ALA, 18:3–9,12,15) is an essential fatty acid, as it cannot be synthesized in human bodies and must be obtained through diets. In humans, ALA is the precursor for the biosynthesis of very long chain ω3-PUFAs, such as eicosapentaenoic acid (EPA, 20:5–5,8,11,14,17) and docosahexaenoic acid (DHA, 22:6–4,7,10,13,16,19). These fatty acids are critical components of membrane glycerolipids and serve as precursors for the bioactive signaling molecules such as eicosanoids and docosanoids regulating various physiological activity in humans and animals (Upchurch, 2008; Mosblech et al., 2009; Yates et al., 2014; Ali et al., 2022; Dyall et al., 2022; Xu et al., 2022).

*Emiliania huxleyi* is a unicellular marine alga belonging to the coccolithophore family found in marine water. It plays important ecological, nutritional and geochemical roles in oceans. The alga has a distinctive structure of coccoliths made of calcium carbonate located in the outer layer of cells with a protection role. After death, the coccoliths sink to the ocean floor, contributing to the long-term storage of carbons in the form of sedimentary rock. Due to its abundance in some regions of oceans, *E. huxleyi* can form massive blooms which can be highly visible from the reflection of coccoliths. These blooms have high impacts on marine ecosystems and carbon cycles (Holligan et al., 1983; Paasche, 2001; Rost and Riebesell, 2004; Hallegraeff and McMinn, 2005). Furthermore, *E. huxleyi* is a primary producer of nutritionally important ω3-PUFAs in the marine food chain. Polyunsaturated fatty acids such as DHA (22:6–4,7,10,13,16,19) and octadecapentaenoic acid (OPA, 18:5–3,6,9,12,15) produced by the alga are a significant source of ω3-PUFAs for a variety of marine organisms in oceans (Bell and Pond, 1996; Leblond et al., 2010; Jónasdóttir, 2019; Graeff et al., 2021).

Two distinct pathways (aerobic and anaerobic) are responsible for the biosynthesis of PUFAs in microorganisms (Qiu et al., 2020). In microalgae, biosynthesis of ω3-PUFAs is generally believed to follow the aerobic pathway, utilizing desaturases to introduce double bonds and elongases to extend acyl chains (Domergue et al., 2002; Petrie et al., 2010). In *E. huxleyi,* several elongases and desaturases in the pathway for the biosynthesis of DHA have been identified. It is assumed that the biosynthesis of DHA in *E. huxleyi* uses ALA as the precursor where ALA is elongated to eicosatrienoic acid (ETA, 20:3–11,14,17) by Δ9 elongase, followed by desaturation catalyzed by Δ8 desaturase, and then another desaturation catalyzed by Δ5 desaturase, giving EPA. EPA can then be elongated by Δ5 elongase to docosapentaenoic acid (DPA, 22:5–7,10,13,16,19), which is then desaturated by Δ4 desaturase to DHA (Sayanova et al., 2011). However, the biosynthesis of OPA still remains elusive in *E. huxleyi* as well as other microalgae.

Omega-3 desaturase is the enzyme introducing an ω3 double bond into ω6 fatty acids, giving corresponding ω3-PUFAs. In another words, it can introduce a Δ15 double bond in C18 linoleic acid (LA, 18:2–9,12) giving ALA, and a Δ17 double bond in C20 arachidonic acid (ARA, 20:4–5,8,11,14) giving EPA. Currently there is substantial information available on ω3 desaturases in plants, fungi and algae involved in the biosynthesis of ω3-PUFAs (Damude et al., 2006; Meesapyodsuk et al., 2007; Chen et al., 2014; Tang et al., 2018; Wu et al., 2021; Klińska-Bąchor et al., 2024). In plants and algae, this kind of enzymes catalyze the synthesis of long chain ω3-PUFAs in both plastids and endoplasmic reticulum (Ohlrogge and Browse, 1995), while in other eukaryotes, the enzymes catalyze the formation of very long chain ω3-PUFAs in endoplasmic reticulum (Pereira et al., 2004; Damude et al., 2006). However, there are limited studies on the ω3 desaturase enzymes that are responsible for the biosynthesis of plastidial ω3-PUFAs in microalgae. In *Chlamydomonas reinhardtii,* two plastidial desaturases (Δ12 and Δ15) were identified that could sequentially introduce Δ12 and Δ15 double bonds in oleic acid (OA, 18:1–9) giving ALA (Sato et al., 1997; Nguyen et al., 2013). In *Ostreococcus tauri*, a Δ15 desaturase was identified that was assumed to function in plastids (Degraeve-Guilbault et al., 2021). In *E. huxleyi*, a Δ15 desaturase was identified, and the function was analyzed by heterologous expression in *Synechocystis* (Kotajima et al., 2014). To the best of our knowledge, no ω3 desaturase with activity to C18- and C20-ω6 fatty acids has ever been identified and functionally characterized in microalgae.

In the present work, we reported molecular cloning and biochemical characterization of a gene (*EhN3*) encoding a plastidial ω3 desaturase in *E. huxleyi*. When expressed in *Saccharomyces cerevisiae*, EhN3 with and without its chloroplast targeting peptide did not exhibit any desaturase activity. However, when expressed in *Synechococcus elongatus*, EhN3 with the chloroplast targeting peptide possessed desaturase activity introducing an ω3 double bond into linoleic acid (LA, 18:2–9,12), γ-linolenic acid (GLA, 18:3–6,9,12), dihomo-γ-linolenic acid (DGLA, 20:3–8,11,14) and arachidonic acid (ARA, 20:4–5,8,11,14) that were exogenously supplied. Lipid analysis of *Synechococcus* transformants fed with C18-ω6 PUFAs showed that both substrates and products were found in monogalactosyldiacylglycerol (MGDG), digalactosyldiacylglycerol (DGDG), indicating these lipids were acyl carrier substrates for the desaturation. In *E. huxleyi*, the transcriptional expression of this gene was regulated by growth temperatures, which was correlated with the production of ω3-PUFAs, particularly OPA.

## Materials and methods

### Strains, plasmids, and culture condition

Axenic *E. huxleyi* strain CCMP1516 was purchased from the National Center for Marine Algae and Microbiota (NCMA) and grown in artificial seawater enriched with the L1 medium minus silicate. The algal cultures were maintained in a 500 mL Erlenmeyer flask under illumination by a LED lamp at an intensity of 100 μmol photons m^−2^ s^−1^ with a light/dark cycle of 12 h/12 h at the optimal temperature 18°C. The growth of the alga was determined based on the optical density (O.D.) at 750 nm. *Synechococcus elongatus* PCC 7942 was purchased from the American Type Culture Collection (ATCC 33912) and cultured in BG-11 medium with shaking at 100 rpm under continuous illumination of 60 μmol photons m^−2^ s^−1^ at 30°C. *Synechococcus* expression vector pSyn6 was obtained from Invitrogen (Carlsbad, CA, USA). The plasmid extraction, DNA purification and gel extraction kits were obtained from Bio Basic (Markham, ON, Canada). Q5 DNA polymerase, restriction enzymes and dNTP were purchased from New England Biolabs (Ipswich, MA, USA). All free fatty acids were purchased from Nu-chek (Nu-chek prep, Elysian, MN, USA).

### Molecular cloning and sequence analysis of ω3 desaturase from *Emiliania huxleyi*

The nucleotide and amino acid sequences of putative ω3 desaturase was retrieved by searching against the published *E. huxleyi* genomic and transcriptome database (Read et al., 2013). Total RNA was isolated from 150 mL culture at the log phase with an RNA extraction Kit (New England Biolab, Ipswich, USA), and 2 μg of total RNA was used to synthesize first-strand cDNA using the SuperScript IV first-strand synthesis system (Invitrogen, Burlington, ON, Canada). To clone the putative ω3 desaturase, 2 μL of the first strand cDNA were used as a template for PCR amplification with two specific primers KS186 and KS187 (Supplementary Table S1). The amplified full-length gene was cloned into a pGEM-T cloning vector (Promega, CA, United States) and sequenced. The sequence of the ω3 desaturase from *E. huxleyi* was analyzed by bioinformatics tools NCBI BLASTp (Camacho et al., 2009) and MegAlign (DNASTAR, Inc. Madison, WI, United States). Three different protein targeting prediction programs were used to predict the putative subcellular locations: PredAlgo,^1^ IPSORT^2^ and TargetP.^3^ Homologous sequences were retrieved from the NCBI for a multi-sequence alignment. Sequence information of EhN3 was deposited in the GenBank under accession number PP273416.

### Transcriptional expression of *EhN3* upon temperature shift

*Emiliania huxleyi* was grown in 300 mL of L1-Si medium at 18°C under the illumination of 100 μmol photons m^−2^ s^−1^ with a light/dark cycle of 12 h/12 h until the culture reached the log phase (O.D.750 at about 0.3). Then, the algal culture was divided into three flasks with a 100 mL aliquot of the culture in each flask. The aliquot cultures were incubated at 11°C, 18°C, and 25°C for 24 h. After that, 80 mL of the cultures were collected by centrifugation at 5,000 rpm and washed twice with fresh medium for RNA extraction, and 20 mL of the cultures were collected for fatty acid analysis. RNA was extracted and treated with DNase I for 30 min to digest possibly contaminated DNA in samples. The first-strand cDNA was synthesized from 1 μg total RNA using SuperScript IV reverse transcriptase for polymerase chain reaction (Invitrogen, Burlington, ON, Canada). The expression of housekeeping gene tubulin was employed as an internal reference and amplified using primer pair KS307 and KS308. The expression level was normalized by that of tubulin. The primer pairs (KS263 and KS299) for PCR were designed using Primer3Plus online software and listed in Supplementary Table S1. The real-time quantitative PCR was performed using PowerUp^™^ SYBR green Master Mix (Fisher Scientific, Carlsbad, CA, USA) according to the manufacturer’s recommendation. Reactions were carried out on a Bio-Rad CFX real-time PCR system (Bio-Rad, Mississauga, Ontario, Canada) as follows: 50°C for 2 min, 95°C for 2 min, 40 cycles of 95°C for 15 s, 60°C for 1 min. Transcriptional expression of EhN3 upon temperature change was analyzed by quantitative RT-PCR and presented as fold change.

### Functional characterization of *EhN3* in *Synechococcus elongatus* PCC 7942

In order to analyze the activity of the ω3 desaturase gene, the full length or cTP-truncated (without first 74 amino acids) open reading frame was inserted into the *Synechococcus* expression vector pSyn6 behind the psbA promoter (Invitrogen, Burlington, ON, Canada). The recombinant plasmids were introduced into the cyanobacterial host for chromosomal integration according to the manual (Invitrogen, Burlington, ON, Canada). *S. elongatus* cells were grown to OD_750_ between 1 to 2, and 1 mL of this culture was washed three times with fresh BG-11 medium and resuspended in 100 μL medium. After that, the recombinant plasmid was added to the suspension, and the mixture was incubated in the dark for 4 h at 34°C and spread on a BG-11 agar plate with 10 μg/mL spectinomycin, which was put under continuous illumination of 50 μmol photons m^−2^ s^−1^ until colonies appeared. After that, colonies were transferred to BG-11 agar plates with elevated concentration of spectinomycin and screened by genomic DNA PCR using forward and reverse primers of Neutral Site 1 (KS212 and KS213). Confirmed *Synechococcus* transformants carrying empty vector, EhN3 and EhN3^Δ^ were then analyzed for the function in the presence of LA, GLA, DGLA and ARA. Appropriate amounts of substrate fatty acids from stocks dissolved in ethanol were exogenously supplied to a 10 mL BG-11 medium culture with OD_750_ at about 1 with the final concentration of 75 μM substrate. The fatty acid-fed culture was grown for 7 days at 22°C with shaking of 100 rpm. Culture samples were collected on day 1, day 2, day 5 and day 7 for lipid and fatty acid analysis (see below).

### Functional analysis of the *EhN3* in *Saccharomyces cerevisiae*

Full length or cTP-truncated open reading frames of ω3 desaturase from *E. huxleyi* were cloned into yeast expression vector pYES2.1 (Invitrogen) under a GAL1 galactose-inducing promoter and introduced into *S. cerevisiae* for heterologous expression. Yeast transformants were selected on synthetic defined agar medium without uracil, and recombinant yeast cells were grown in the same medium at 28°C for 2 days. The culture was then washed and diluted in 10 mL induction medium and supplemented free fatty acids LA and GLA at the final concentration of 250 μM individually in the presence of 0.1% Tergitol. The induced culture was grown for another 2 days at room temperature, washed with 1% Tergitol and sterile water, and then used for fatty acid analysis.

### Lipid analysis of cyanobacterial transformants expressing *EhN3*

Alga biomass from cultures was collected by centrifugation at 8000 rpm for 5 min, washed with 0.1 M NaHCO_3_. Total lipids of cyanobacterial transformants expressing full length or cTP-truncated EhN3 were extracted with methanol/chloroform/water after homogenization with a glass rod. Total lipids were separated primarily into monogalactosyldiacylglycerol (MGDG), digalactosyldiacylglycerol (DGDG) and phosphatidylglycerol (PG) on thin layer chromatography (TLC) plates using acetone/toluene/water (91,30:8, v/v/v). Individual lipid classes were identified by the comparison with known commercial standards (Abitec, Monroe, MI, USA; Sigma-Aldrich, St. Louis, MO, USA) and scraped off the plates for fatty acid composition analysis. Lipid classes were directly transmethylated to fatty acid methyl esters (FAMEs) by heating at 85°C for 1 h with 2% sulfuric acid in methanol after being scraped from TLC plates.

### Fatty acid analysis

Fatty acids in yeast and cyanobacterial transformants were analyzed by gas chromatography with heptadecanoic acid (17:0) as the internal standard. Total fatty acids in the biomass were transmethylated to fatty acid methyl esters (FAMEs) by 2 mL of 1% sulfuric methanol at 80°C for 2 h. After transmethylation, the samples were cooled down on the ice and then added with 1 mL 0.9% NaCl and 2 mL hexane. After brief vertexing and then centrifugation, the hexane phase containing FAMEs was transferred to a new tube and dried under N_2_ gas. After drying, the sample was re-suspended in an appropriate amount of hexane and used for GC analysis on an Agilent 7890A system equipped with a DB-23 column (30 m × 0.25 mm) with 0.25 μm film thickness (J&W Scientific, Folsom, CA, USA) with a temperature program of 180°C for 1 min, 4°C/min to 240°C, hold for 15 min. The new peaks shown on the GC chromatogram were compared with Supelco 37-component standards (Sigma-Aldrich, St. Louis, MO, USA) (Xie et al., 2020).

## Results

### Molecular cloning and sequence analysis of an ω3 desaturase gene from *Emiliania huxleyi*

**Figure 1:**
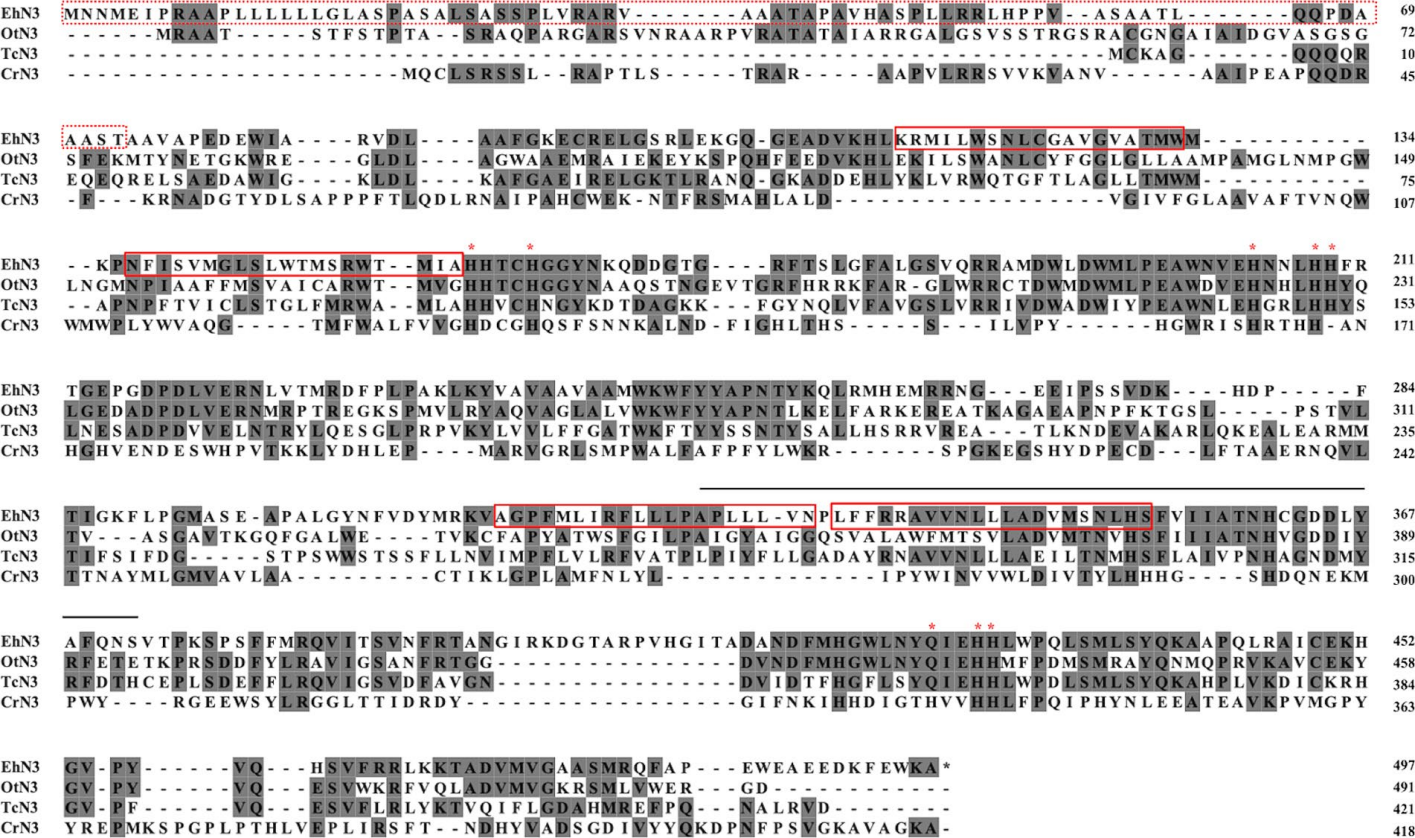
Multiple sequence alignment of EhN3 and its homologous Δ15/ω3 desaturases from algae. The predicted chloroplast transit peptide (cTP) of EhN3 were enclosed in a red dashed box, and four predicted transmembrane domains were enclosed by red solid boxes. Conserved histidine/glutamine residues of three histidine boxes were indicated by asterisk (*). The extra 48 amino acids over the previously identified protein EOD40666 (Kotajima et al., 2014) were shown with a solid line on top. Accession numbers of the three functionally characterized homologs included in the alignment: OtN3 (OUS43900.1), *Ostreococcus tauri* fatty acid desaturase (Degraeve-Guilbault et al., 2021); TcN3 (AOG21010), *Thraustochytrium* sp. ATCC 26185 omega 3 desaturase (Meesapyodsuk and Qiu, 2016); CrN3 (EDP09401.1), *Chlamydomonas reinhardtii* omega 3 desaturase (Nguyen et al., 2013).

To clone the gene encoding ω3 desaturase from *E. huxleyi*, functionally characterized ω3 desaturases from protists and fungi were used as queries to search the genome and transcriptome databases of *E. huxleyi* (JGI Project ID: 16965).^4^ A candidate gene named as *EhN3* was identified by the homologous search. A pair of specific primers targeting outside of the open reading frame were then designed and used for reverse transcription PCR with the total RNA as template for amplifying the gene. Sanger sequencing of the full-length cDNA (accession number in NCBI: PP273416) revealed an open reading frame of 1,491 bp encoding a polypeptide of 496 amino acids, which was 48 amino acids longer than the one previously reported (Kotajima et al., 2014). A long chloroplast transit peptide (cTP) was found in the N-terminal sequence, suggesting it is probably targeted to algal plastids. Amino acid sequence alignment of EhN3 with the algal homologs showed that all these proteins possessed three histidine motifs, a typical feature of the desaturases. Like front-end desaturases, the third histidine motif (Q-I-E-H-H) of EhN3 contained glutamine instead of histidine (Shanklin et al., 1994; Sayanova et al., 1997; Meesapyodsuk and Qiu, 2012). The additional 48 amino acids of EhN3 were located in the middle region with a transmembrane helix (TM4) where a histidine (His361) was situated for coordinating an iron modeled by AlphaFold2 (Figure 1 and Supplementary Figure S1). This result suggests this region might be essential for the function of the desaturase.

### Functional analysis of EhN3 in *Synechococcus elongatus*

**Figure 2:**
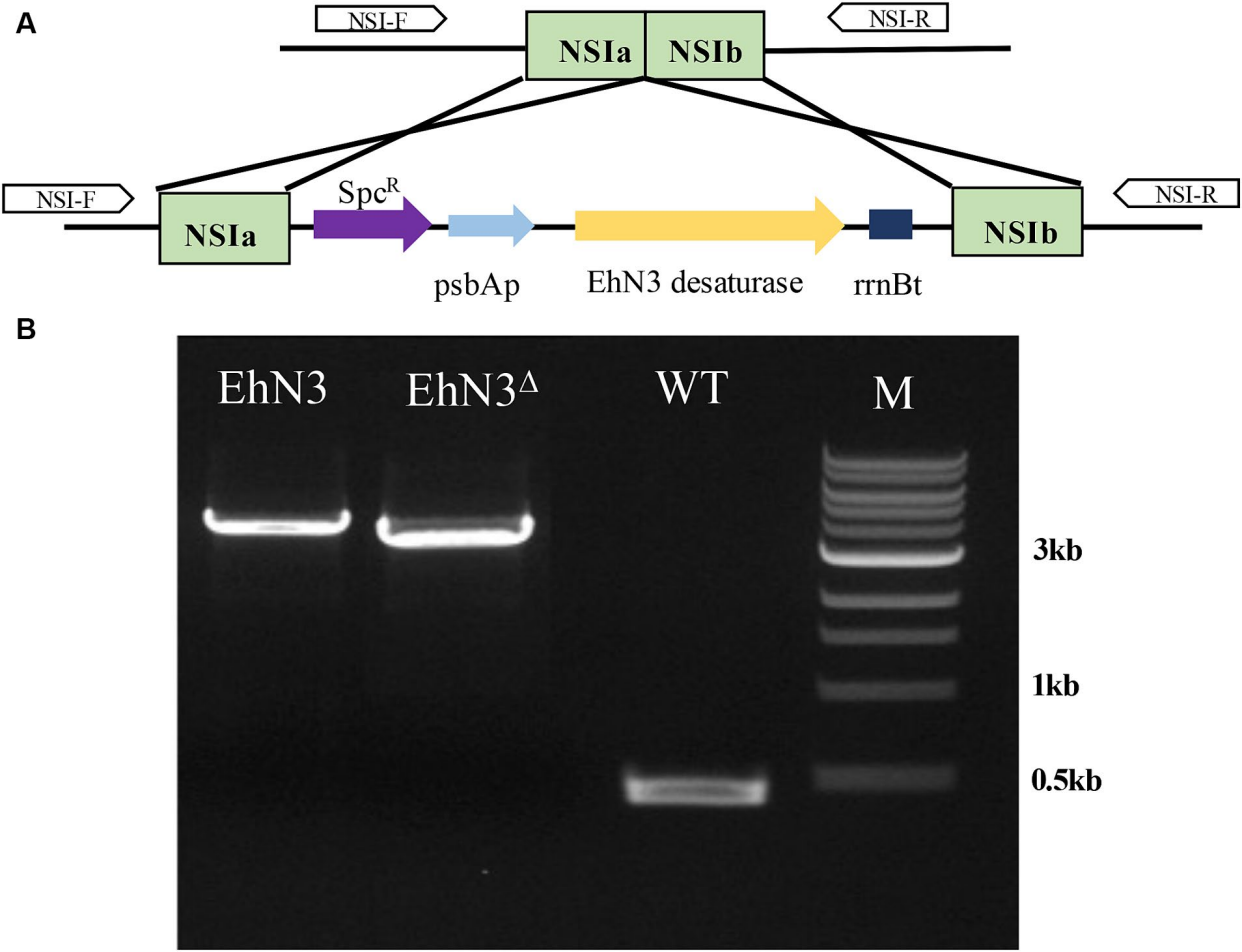
Integration of the EhN3 expression cassette into *Synechococcus elongatus* PCC 7942. **(A)** Schematic illustration of the integration. **(B)** Electrophoresis of colony PCR amplification fragments from transformants with EhN3 and EhN3^Δ^ confirming the integrations. WT, wild type; M, DNA size marker.

To analyze the function of EhN3, a cyanobacterium strain *Synechococcus elongatus* PCC 7942 was first exploited to express the gene. The full open reading frame was cloned into a *Synechococcus* expression vector under the control of psbA promoter and rrnB terminator, and the recombinant plasmid was transformed into the *Synechococcus* strain by natural transformation. Integration of the *EhN3* expression cassette into the cyanobacterial genome by homologous recombination was confirmed by PCR amplification of an about 3.7 kb fragment using genomic DNA as template and primers flanking the insertion sites (NSIa and NSIb) (Figure 2).

**Figure 3:**
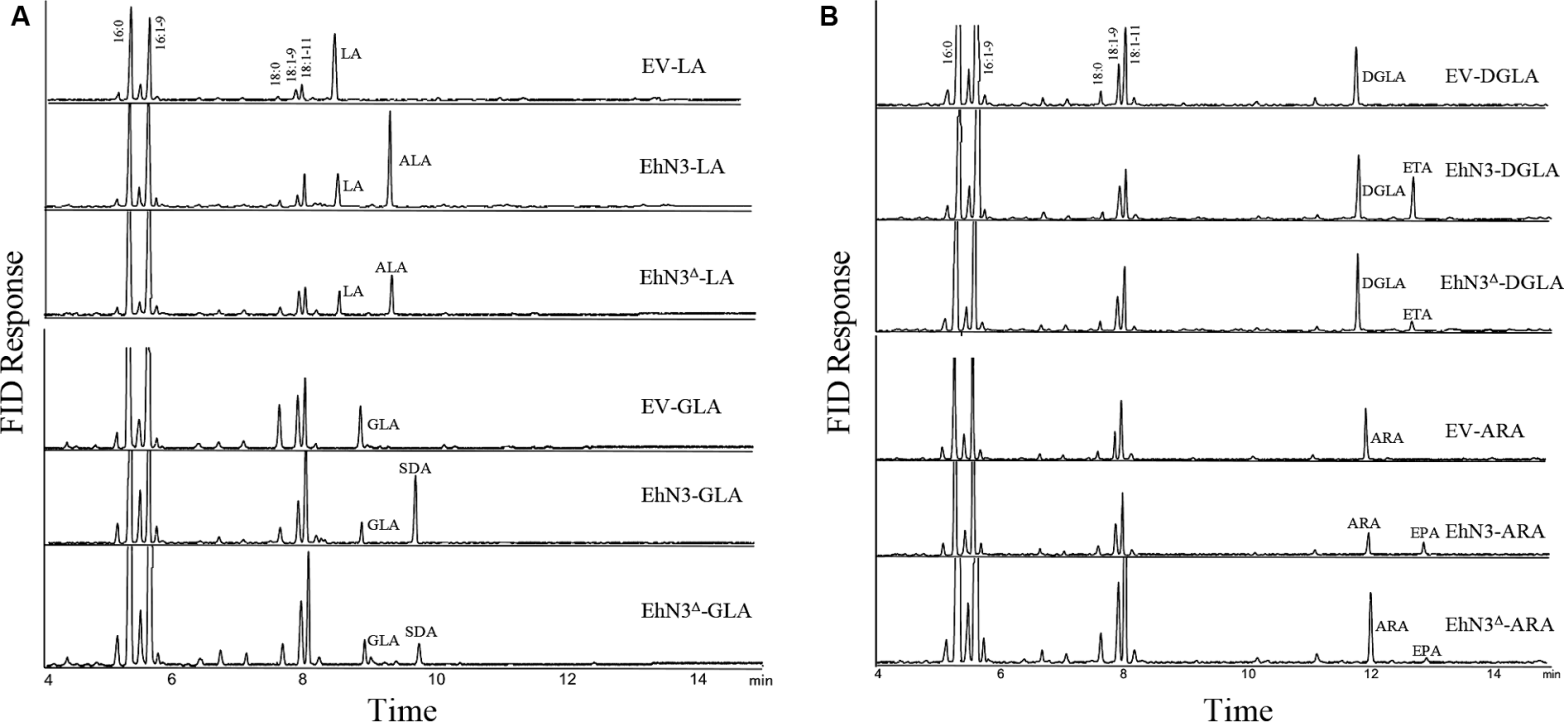
Fatty acid profiles of *Synechococcus* transformants expressing EhN3 and EhN3^Δ^ fed with C18 and C20 substrates. **(A)** GC analysis of FAMEs prepared from *Synechococcus* transformant with empty vector, EhN3 and EhN3^Δ^ fed with 18 carbon chain substrates linoleic acid (LA) and γ-linolenic acid (GLA). EV-LA, *Synechococcus* expressing empty vector pSyn6 fed with LA; EhN3-LA, *Synechococcus* expressing full length EhN3 fed with LA; EhN3^Δ^-LA, *Synechococcus* expressing EhN3 without cTP fed with LA; EV-GLA, *Synechococcus* expressing empty vector pSyn6 fed with GLA; EhN3-GLA, *Synechococcus* expressing full length EhN3 fed with GLA; EhN3^Δ^-GLA, *Synechococcus* expressing EhN3 without cTP fed with GLA. **(B)** GC analysis of FAMEs prepared from *Synechococcus* transformant with empty vector, EhN3 and EhN3^Δ^ fed with 20 carbon chain substrates dihomo- γ-linolenic acid (DGLA) and arachidonic acid (ARA). EV-DGLA, *Synechococcus* expressing empty vector pSyn6 fed with DGLA; EhN3-DGLA, *Synechococcus* expressing EhN3 fed with DGLA; EhN3^Δ^-DGLA, *Synechococcus* expressing EhN3 without cTP fed with DGLA; EV-ARA, *Synechococcus* expressing empty vector pSyn6 fed with ARA; EhN3-ARA, *Synechococcus* expressing EhN3 fed with ARA; EhN3^Δ^-ARA, *Synechococcus* expressing EhN3 without cTP fed with ARA.

To analyze ω3 desaturase activities, the confirmed transformants were grown in the presence of C18 substrate fatty acids LA and GLA, respectively. As compared with *Synechococcus* wild type transformed with the empty vector (control) fed with LA, transformants expressing EhN3 fed with LA produced a new peak with a retention time identical to that of ALA. The transformant fed with GLA produced a new peak with a retention time identical to that of stearidonic acid (SDA, 18:4–6,9,12,15) (Figure 3A). To investigate whether this desaturase possessed ω3 desaturase activity toward very long chain ω6-PUFAs, dihomo-γ-linolenic acid (DGLA, 20:3–8,11,14) and arachidonic acid (ARA, 20:4–5,8,11,14) were supplied exogenously to the transformants. Fatty acids analysis showed that transformant produced a new peak with retention time identical to that of eicosatetraenoic acid (ETA, 20:4–8,11,14,17) in the presence of DGLA, and produced a new peak with retention time identical to that of EPA (20:5–5,8,11,14,17) in the presence of ARA, as compared to the control (Figure 3B). These results clearly indicate that *EhN3* from *E. huxleyi* codes for a fatty acid ω3 desaturase which can introduce a Δ15 double bond into both LA and GLA, giving their corresponding ω3 fatty acids, and introduce a Δ17 double bond into both DGLA and ARA, giving their corresponding ω3 fatty acids.

To investigate if the transit peptide at the N-terminus of EhN3 was required for function, the open reading frame with the chloroplast targeting peptide (cTP) being removed was cloned into the same *Synechococcus* expression vector and introduced into the *Synechococcus* strain. Fatty acid analysis showed that transformants expressing EhN3 without cTP (EhN3^Δ^) exhibited the similar function as the full open reading frame, i.e., producing ALA and SDA in the presence of LA and GLA, respectively, as well as ETA and EPA in the presence of DGLA and ARA, respectively, although conversion efficiency of C20 substrates was lower than that of C18 substrates (Figures 3A,B). These results indicate that the signal peptide for chloroplast targeting at the N-terminus of EhN3 does not essentially affect the functionality of EhN3 when expressed in *Synechococcus*.

### Functional analysis of EhN3 in *Saccharomyces cerevisiae*

To examine whether EhN3 was also functional in yeast, the full open reading frame and that without the signal peptide were individually cloned into a yeast expression vector under the control of an inducible promoter, and the recombinant plasmids were transformed into *S. cerevisiae* INVSc1 strain. Fatty acid analysis showed that the control yeast transformed with the empty vector produced four major fatty acids, 16:0, 16:1–9, 18:0 and 18:1–9, and both transformants expressing EhN3 with and without the transit peptide did not produce any new fatty acids regardless of the presence and absence of LA, GLA, DGLA and ARA, while the positive control was well functional (Supplementary Figure S2 for LA supplementation). This result implies EhN3 might not exhibit any desaturase activity when expressed in yeast *S. cerevisiae*.

### Acyl carrier substrate of EhN3 in *Synechococcus*

**Figure 4:**
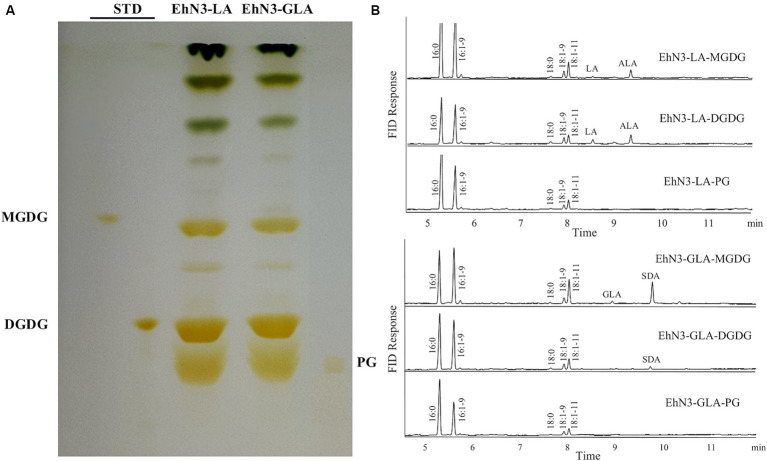
Lipid analysis of *Synechococcus* transformants expressing EhN3 desaturase fed with LA and GLA. **(A)** Lipids were separated by thin layer chromatography and visualized by iodine vapor. **(B)** Fatty acid analysis of MGDG, DGDG and PG from transformants fed with LA (upper) and GLA (lower) that were scrapped from TLC plate and analyzed by GC.

Algal desaturases usually have the strict requirement for substrate forms depending on their locations. Extraplastidial endoplasmic reticulum desaturase would use phosphoglycerolipids as substrates while plastidial membrane desaturases would use glycoglycerolipids as substrates. To examine the acyl carrier substrates for EhN3, the total lipids were extracted from the *Synechococcus* transformants grown in the presence of LA and ALA, and then fractionated by thin layer chromatography (TLC). Major lipid classes in the cyanobacteria such as monogalactosyldiacylglycerol (MGDG), digalactosyldiacylglycerol (DGDG) and phosphatidyl glycerol (PG) were scrapped off from the TLC plate and transmethylated for fatty acid analysis. As shown in Figure 4, when LA and GLA were fed to the transformant expressing EhN3, they were effectively incorporated into glycoglycerolipids MGDG and DGDG where they were desaturated to ALA and SDA, respectively. However, there was no incorporation of exogenously fed LA and GLA into PG, and no desaturation product was found in the phospholipid. This result implies that EhN3 utilizes glycoglycerolipid MGDG and DGDG as substrates for desaturation.

### A time course of desaturation efficiency of EhN3 in *Synechococcus*

**Figure 5:**
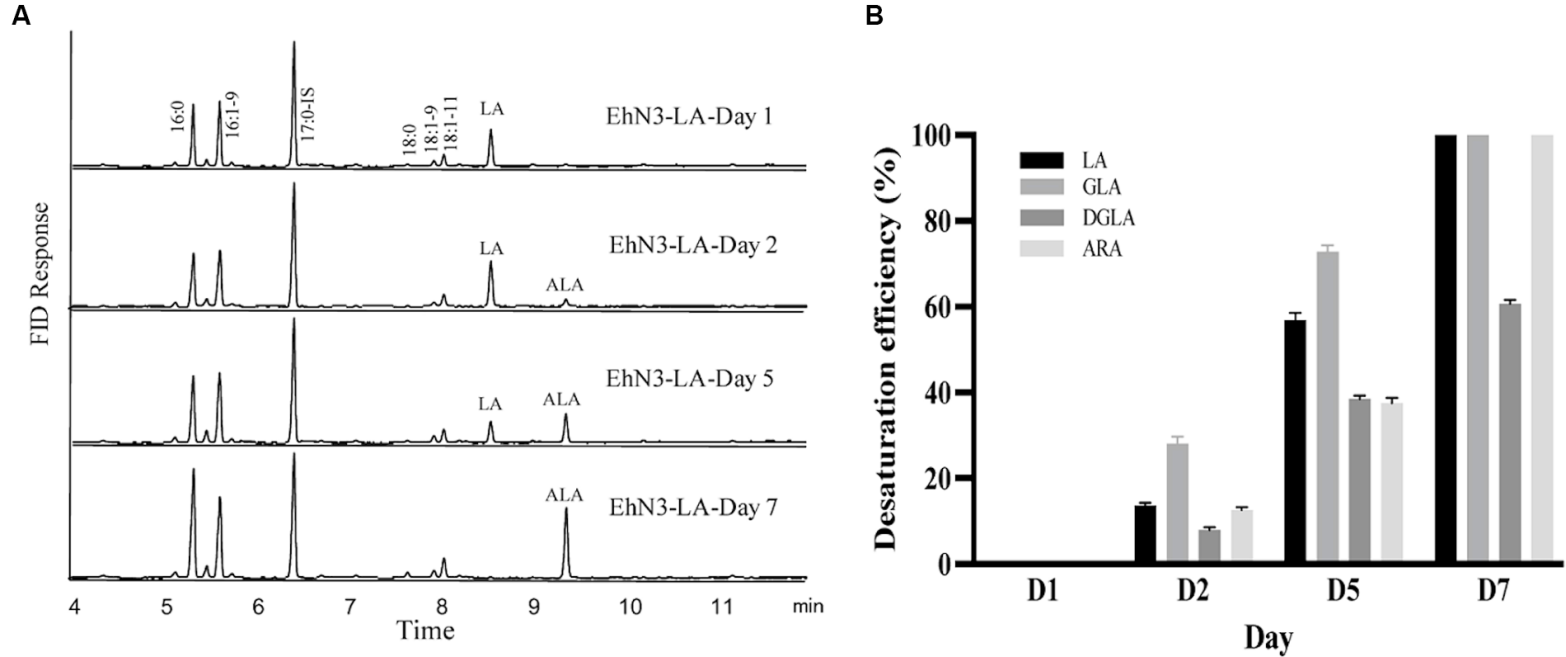
Fatty acid analysis of *Synechococcus EhN3* transformants grown in presence of LA, GLA, DGLA and ARA in a time course. **(A)** GC analysis of FAMEs prepared from transgenic *Synechococcus* expressing *EhN3* fed with LA on Day 1, 2, 5 and 7. **(B)** Desaturation efficiency of *Synechococcus* transformants on LA, GLA, DGLA and ARA on Day 1, 2, 5 and 7 calculated through a formula: product/(substrate + product) ×100%. Data were collected from 3 individual colonies with each colony repeating three times.

Next, a seven-day time course of EhN3 and EhN3^Δ^ desaturation activity was investigated in *Synechococcus* fed with both C18 and C20 substrates, and fatty acid profiles of transformants expressing both forms on Day 1, Day 2, Day 5 and Day 7 were analyzed. As shown in Figure 5, the desaturation efficiency increased during the time course. In *Synechococcus* transformant expressing the full-length EhN3 fed with LA, the desaturation efficiency of LA to ALA increased from almost nothing on Day 1 to about 100% on Day 7. In the transformant fed with GLA, the desaturation efficiency of GLA to SDA also increased from nothing on Day 1 to about 100% on Day 7. Similarly, when *Synechococcus* transformant expressing EhN3 fed with ARA, the desaturation efficiency of ARA to EPA was zero on Day 1, and reached about 100% on Day 7. In the transformant fed with DGLA, the desaturation efficiency increased from almost nothing on Day 1 to about 61% on Day 7. These results indicate EhN3 is a highly active ω3 desaturase that can effectively convert both C18-ω6 and C20-ω6 fatty acids to their corresponding ω3 fatty acids in a time course.

### Transcriptional expression of EhN3 and fatty acid composition in *Emiliania huxleyi* at different temperatures

**Figure 6:**
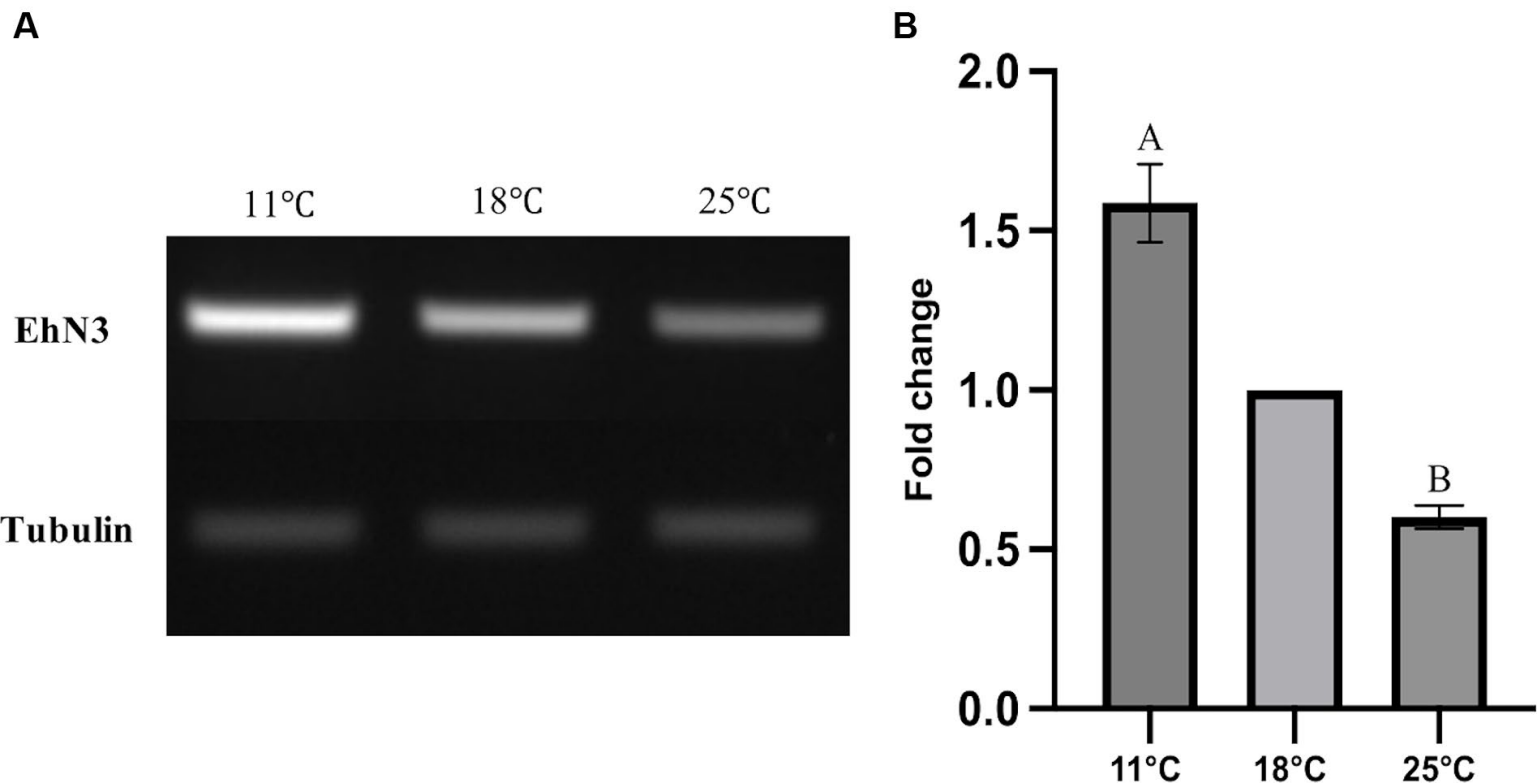
Transcriptional expression analysis of *EhN3* in *E. huxleyi* at three different temperatures (11°C, 18°C, and 25°C). **(A)** The mRNA levels of endogenous EhN3 and reference tubulin genes were analyzed by semi-quantitative PCR and visualized by electrophoresis. **(B)** The mRNA levels of endogenous EhN3 and reference tubulin genes were analyzed by quantitative PCR and visualized by fold changes. The expressions were first normalized upon the expression level of the housekeeping gene. The fold changes were then calculated relative to the expression level at 18°C which was set to 1. Values were reported as the means of three biological replicates along with standard deviation. The means with the same letters are not statistically significantly different. Statistical analysis of the results was conducted using the one-way analysis of variance (*p* < 0.05).

It is well known that temperature possesses significant impacts on cell membrane fluidity modulated by fatty acid desaturases in algae and plants (Kodama et al., 1995; Horiguchi et al., 2000; Falcone et al., 2004; Guschina and Harwood, 2006; Los et al., 2013; Nalley et al., 2018). To examine whether the temperature change has any effects on the expression of *EhN3* in *E. huxleyi*, the transcriptional expression of this gene in the alga grown under 11°C, 18°C, and 25°C for 24 h was determined by semi-quantitative and full quantitative RT-PCR analysis. Both results similarly showed that the growth temperature impacted the transcriptional expression of this gene. This was initially reflected in semi-quantitative PCR analysis where the expression level of *EhN3* was sequentially increased in the alga grown from 25°C to 18°C, and to 11°C. From the full quantitative PCR analysis, the expression level of *EhN3* in the microalga grown at 11°C was increased by 1.6-fold relative to that grown at 18°C. When the microalga was grown at 25°C, the transcript level of *EhN3* was decreased by 0.6-fold relative to that grown at 18°C (Figure 6). It was also noted that the transcript change at different temperatures was correlated with the composition of some ω3 fatty acids such as OPA, SDA and ALA. The total amount of ω3 fatty acids was higher when the microalga grown at 11°C than those grown at 18°C and 25°C. Particularly, the amount of OPA in the alga grown at 11°C was significantly increased over those at 18°C and 25°C (Table 1).

**Table 1:** Fatty acids compositions (%) of *E. huxleyi* cultures under different temperatures.

Fatty acid (%)	14:0	16:00	18:1–9	18:1–11	ALA	SDA	OPA	DHA
11°C	13.08 ± 0.09^b^	7.46 ± 0.12^b^	9.74 ± 0.15^b^	3.19 ± 0.06^ab^	8.64 ± 0.31^a^	11.01 ± 0.15^a^	26.68 ± 0.18^a^	17.93 ± 0.18^b^
18°C	13.22 ± 0.43^b^	7.02 ± 0.27^b^	10.25 ± 0.06^a^	3.14 ± 0.06^b^	8.04 ± 0.20^b^	10.76 ± 0.12^a^	25.75 ± 0.17^b^	19.58 ± 0.18^a^
25°C	15.63 ± 0.18^a^	8.98 ± 0.19^a^	9.74 ± 0.17^b^	3.41 ± 0.05^a^	7.62 ± 0.08^c^	11.19 ± 0.24^a^	23.55 ± 0.12^c^	17.75 ± 0.20^b^

Values were reported as the mean of four biological replicates with standard deviation. The means for each value in a single column with different letters are statistically significantly different from each other according to the one way ANOVA analysis (*p* < 0.05).

## Discussion

Microalgae have gained much attention in recent years due to their potential for the production of biofuel and high value bioproducts in response to concerns about the limitations and environmental impact of fossil fuel (Liu and Benning, 2013). *E. huxleyi* is a unique microalga that plays an important ecological and geochemical role due to its distinctive structure and chemical nature of coccoliths and abundance in oceans. In addition, it also plays significant nutritional role in oceanic food chain as it can produce a variety of ω3 polyunsaturated fatty acids including ALA (18:3n-3), SDA (18:4n-3), OPA (18:5n-3) and DHA (22:6n-3). However, the biosynthesis of these fatty acids in the alga is not well understood. Previously, Sayanova et al. (2011) identified five genes encoding endoplasmic reticulum Δ4 desaturase, Δ5 desaturase, Δ8 desaturase, Δ9 elongase and Δ5 elongase, suggesting DHA might be synthesized from the precursor ALA by an extraplastidial aerobic pathway in this alga. However, the genes encoding Δ12 and Δ15 desaturases in the pathway for the biosynthesis of ALA from precursor 18:1–9 have not been identified. In addition, no enzymes involved in the biosynthesis of OPA in the plastidial aerobic pathway have been identified. Kotajima et al. (2014) identified a gene in *E. huxleyi* encoding plastidial Δ15 desaturase involved in the biosynthesis of ALA, a precursor for OPA biosynthesis in plastids, and the function of this enzyme with a missing transit peptide at the N-terminus was determined by heterologous expression in *Synechocystis* where several PUFAs such as SDA, GLA and ALA are produced. In this study, a plastidial ω3 desaturase gene (*EhN3*) was cloned from the same alga species, and the function of the gene with and without the transit peptide were characterized by heterologous expression in *Synechococcus* where no PUFAs are produced. Our comparison of the two sequences surprisingly revealed 48 amino acids extra in EhN3 over the one previously identified (Kotajima et al., 2014). These extra amino acids are located in the middle region of the protein ranging from residue 326 to 373, including one transmembrane helix (Figure 1 and Supplementary Figure S1). Membrane desaturases usually comprise four transmembrane helices which are essential for defining the architecture of the substrate tunnel for substrate acceptance and interaction. Therefore, it is surprising to see that the previously identified one with missing 48 amino acids was still functional when expressed in *Synechocystis*.

In plants and algae, there are two different types of Δ15/ω3 desaturases with distinct locations and substrates for the biosynthesis of ALA in phosphoglycerolipids and glycoglycerolipids (Ohlrogge and Browse, 1995). In contrast to higher plants, there are limited studies on the structure and function of the ω3 desaturases in microalgae. In this study, a functional plastidial ω3 desaturase was identified in *E. huxleyi* that produces a substantial amount of ω3 PUFAs such as OPA in plastidial glycoglycerolipids. The function of the desaturase was determined by expressing it in *Synechococcus elongatus*, a freshwater unicellular cyanobacterium that has been used as a prokaryotic photosynthetic model system. Unlike *Synechocystis* that was used in a previous study (Kotajima et al., 2014) where there is an endogenous Δ15 desaturase for producing ALA, *Synechococcus elongatus* does not contain Δ12 and Δ15 desaturases and produces only monounsaturated fatty acids 16:1–9 and 18:1–9. Thus, it is a better system for functional analysis of membrane desaturases for PUFA biosynthesis, as it would avoid the interference of endogenous desaturase activity. The ω3 desaturase activity of EhN3 was established by the expression in *Synechococcus* fed with C18-ω6 fatty acids LA and GLA as well as C20-ω6 fatty acids DGLA and ARA, and transformants produced corresponding C18-ω3 fatty acids ALA and SDA, and C20-ω3 fatty acids ETA and EPA, respectively. In addition, the desaturation activity was observed to increase during a time course where desaturation efficiency started from almost nothing to about 100% on C18 substrates LA and GLA as well as C20 substrate ARA. Similar high ω3 desaturase activity toward both C18 and C20 substrates has never been seen in alga desaturases. In a study where an active plastidial Δ12 fatty acid desaturase from *Phaeodactylum tricornutum* was expressed in *Synechococcus*, the desaturation efficiency was 87% on the substrate 18:1–9 (Domergue et al., 2003). Lipid analysis of *S. elongatus* transformants expressing EhN3 indicated that desaturation products were accumulated only in glycolipid MGDG and DGDG, but not in phospholipid PG. This result, along with the facts that it contains a chloroplast targeting peptide at the N-terminus and is functional in *Synechococcus,* but not in yeast, implies that EhN3 is directed by the transit peptide to chloroplasts of *E. huxleyi* where it utilizes galactolipid as acyl carrier substrate for desaturation.

EhN3 and cyanobacterium desaturases share some common structural features such as histidine boxes for coordinating metal irons and membrane-associated domains for integration into thylakoid membranes, as both are responsible for introducing double bonds in fatty acyl chains of glycoglyerolipids. As compared to microalgae with a wide range of fatty acids from 14 to 22 carbon chains and one to six double bonds, cyanobacteria have rather limiting sets of fatty acids, ranging from 14 to 18 carbon chains with one to four double bonds (Los and Mironov, 2015). In *Synechococcus* sp. PCC 7942 that produces only monounsaturated fatty acids, a single Δ9 desaturase gene is found in the genome. In *Synechococcus* sp. PCC 7002 and *Anabaena* sp. PCC 7120 that can produce ALA, Δ9, Δ12 and Δ15 desaturases are used to synthesize the ω3 fatty acid. In *Spirulina* sp. that produces GLA, Δ6, Δ9 and Δ12 desaturases are used to synthesize the ω6 fatty acid. In *Synechocystis* sp. PCC 6803 that can produce stearidonic acid (SDA, 18:4–6,9,12,15), four desaturases (Δ6, Δ9, Δ12 and Δ15) are responsible for the synthesis of this highly unsaturated 18C chain fatty acid (Murata et al., 1992; Xiaoyuan et al., 2008). However, unlike cyanobacterium desaturases, EhN3 contains a chloroplast-targeting peptide (cTP). Whether the signal peptide is required for the function in *Synechococcus* could be questionable, as there is no chloroplast envelop membrane inside a *Synechococcus* cell, but the transit peptide might subtly influence the folding structure of the mature enzyme. However, when the signal peptide was removed from the full length ω3 desaturase (EhN3^Δ^), it still functioned in *Synechococcus* as did the full-length desaturase (EhN3) with ω3 desaturation activity toward both C18 and C20 substrates, although activity to these substrates was decreased. This result is informative for heterologous expression of eukaryotic plastidial enzymes in cyanobacteria where the signal peptide of the enzymes for chloroplast-targeting may not be required. On the other hand, when EhN3^Δ^ was expressed in yeast, it did not exhibit any desaturation activity probably due to its lack of thylakoid structure. However, there are reports that plastidial desaturases with or without cTP are functional in yeast (Domergue et al., 2003; Liu et al., 2020). Plastidial desaturases utilize ferredoxin/NADPH-ferredoxin reductase as electron donors, while microsomal desaturases use cytochrome b5/NADH-cytochrome b5 reductase as electron donors for desaturation (Meesapyodsuk and Qiu, 2012). The function of some plastidial desaturases in yeast implies that the electron donors can be shared to some extent with different types of desaturases (Domergue et al., 2003). However, non-functionality of EhN3 with or without cTP in yeast, although the possibility that EhN3 and EhN3^Δ^ might not be properly expressed at the protein level cannot be absolutely excluded, would suggest that either specific electron donor and/or acyl-carrier substrate might be strictly required for the activity. Similar phenomenon was also observed with two plastidial Δ6 desaturases from *Ostreococcus tauri* and a plastidial Δ15 desaturase from alga *Chromochloris zofingiensis* (Degraeve-Guilbault et al., 2020; Wu et al., 2021).

It is well documented in literature that expressions of desaturases are modulated by temperature to meet the dynamic requirement of cellular membrane fluidity in cyanobacteria, algae and plants (Sakamoto and Bryant, 1997; Degenkolbe et al., 2012; Román et al., 2012; Los et al., 2013; Los and Mironov, 2015; Nalley et al., 2018; Willette et al., 2018; Degraeve-Guilbault et al., 2021). Unsaturated fatty acids were also found important in the tolerance to salt stress (Allakhverdiev et al., 1999). Increasing the degree of fatty acid unsaturation not only helps cyanobacteria tolerate low temperature by altering the fluidity of thylakoid membrane, but also accelerates the recovery process of photosynthesis after temperature-induced photoinhibition (Murata and Wada, 1995; Ludwig and Bryant, 2012). In addition, heterologous expression of a cyanobacterial desaturase significantly enhances low-temperature resistance in higher plants (Ishizaki-Nishizawa et al., 1996). In the present study, the variation of the EhN3 expression and fatty acid compositions in *E. huxleyi* were also observed in response to temperature changes. The transcriptional expression of *EhN3* was increased by 1.6-fold at 11°C and decreased by 0.6-fold at 25°C relative to that at 18°C. The expression pattern was correlated with some ω3 fatty acid production in the microalga grown under the temperatures. Particularly, the amount of OPA was significantly increased in the alga when grown from 25°C to 18°C, and to 11°C. The increased OPA could be beneficial in the acclimation of this microalga to cold temperature. These results therefore indicate that EhN3 might play a role in the biosynthesis of OPA in plastids and modulating the membrane fluidity of this alga in response to temperature changes in oceans.

## Data availability statement

The datasets presented in this study can be found in online repositories. The names of the repository/repositories and accession number(s) can be found in the article/Supplementary material.

## Author contributions

KS: Data curation, Formal analysis, Methodology, Writing – original draft, Writing – review & editing. DM: Investigation, Supervision, Writing – review & editing. XQ: Funding acquisition, Investigation, Project administration, Resources, Supervision, Writing – review & editing.

## Footnotes

^1^ https://lobosphaera.ibpc.fr/cgi-bin/predalgodb2.perl?page=main

^2^ https://ipsort.hgc.jp/index.html

^3^ https://services.healthtech.dtu.dk/services/TargetP-2.0

^4^ https://phycocosm.jgi.doe.gov/Emihu1/Emihu1.home.html
