# Supplementary figures and images for: Molecular cloning and functional analysis of a plastidial ω3 desaturase from Emiliania huxleyi
Source: Front Microbiol. 2024 Jul 11;15:1381097. doi: 10.3389/fmicb.2024.1381097 (PMC11269151; doi:10.3389/fmicb.2024.1381097)

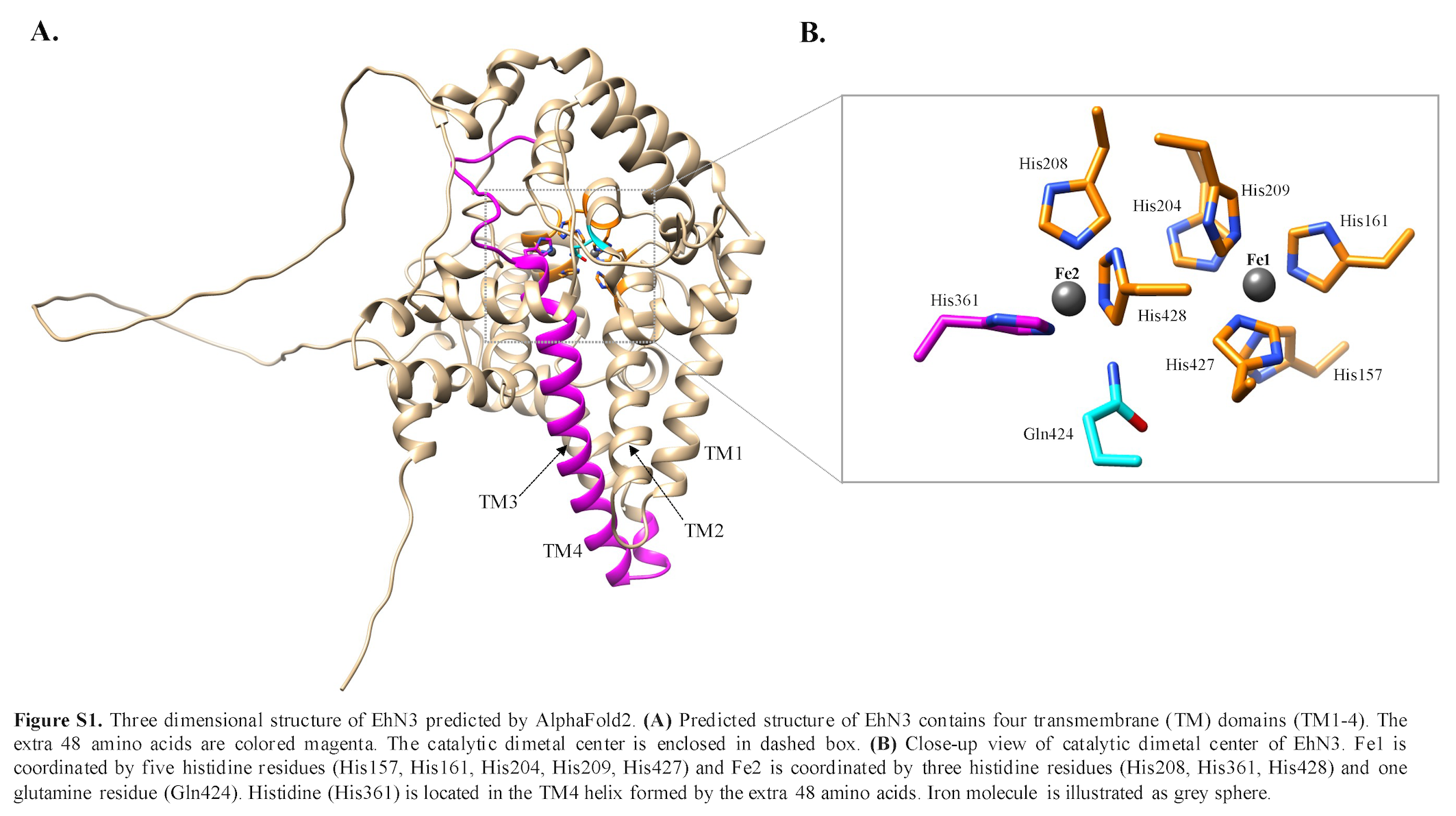

Supplement: Supplementary file 1 [file Image_1.TIFF]

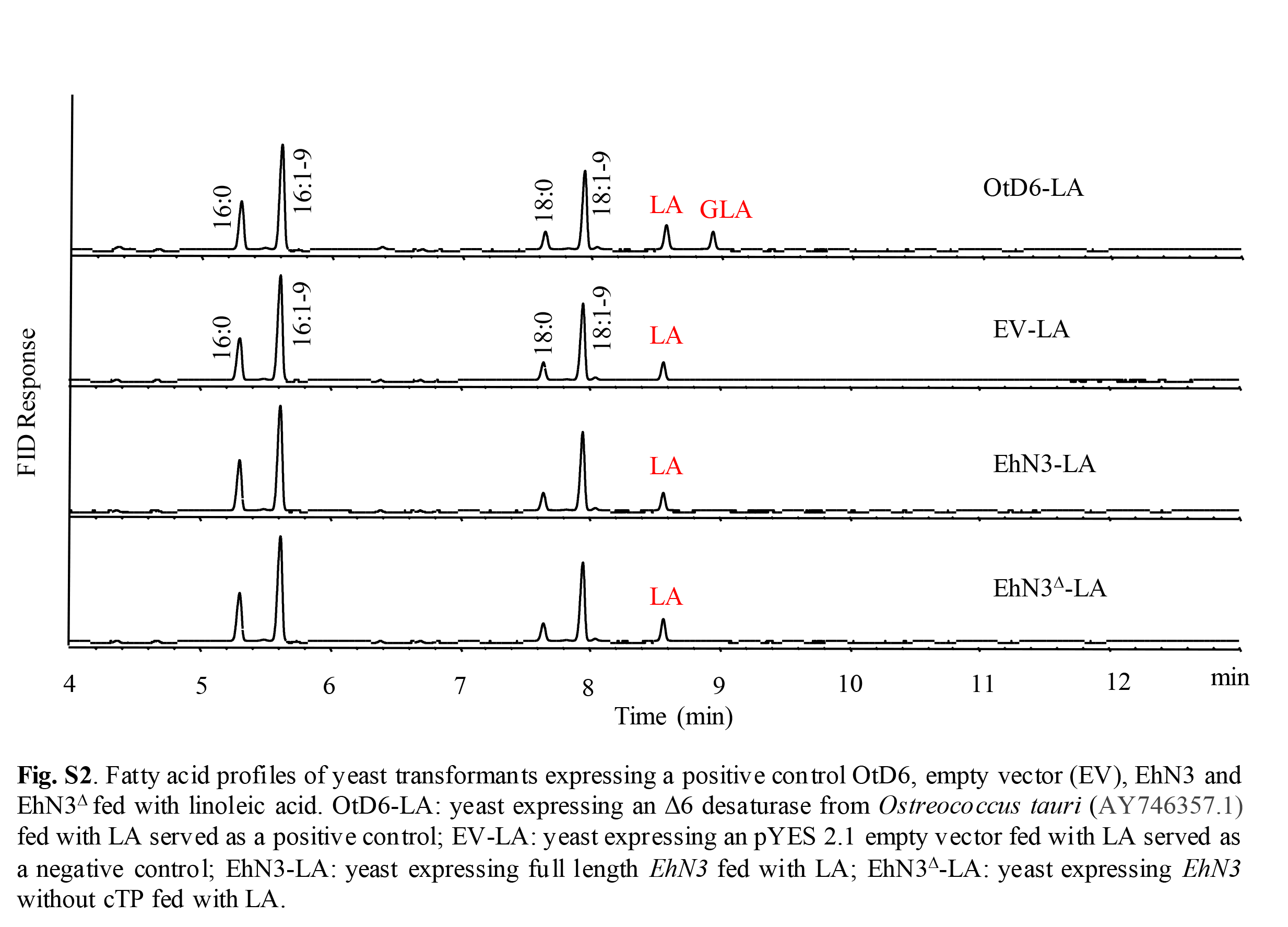

Supplement: Supplementary file 2 [file Image_2.TIF]
