# Supplementary material for: Molecular cloning and functional analysis of a plastidial ω3 desaturase from Emiliania huxleyi
Source: Front Microbiol. 2024 Jul 11;15:1381097. doi: 10.3389/fmicb.2024.1381097 (PMC11269151; doi:10.3389/fmicb.2024.1381097)
Supplement: Supplementary file 3 [file Table_1.DOCX]

| **Primer** | **Sequence (5’-3’)** | **Annotation** |
| --- | --- | --- |
| KS186 | ATGAACAACATGGAGATCCC | Gene specific-F |
| KS187 | CTACGCCTTCCACTCGAAC | Gene specific-R |
| KS 212 | AGGAGCGGATCAAACAGGT | Flanking Neutral site I-F |
| KS213 | TATGGTTCGGGATCACTGG | Flanking Neutral site I-R |
| KS263 | CCTGAAGCGCATGATCCTCT | EhN3-qRT-PCR-F |
| KS299 | ATCGTGACGAGGTTACGCTC | EhN3-qRT-PCR-R |
| KS307 | CGCTGTACGACATCTGCTT | Eh-Tubulin-qRT-PCR-F |
| KS308 | GGAAGGGGATCATGTTGAC | Eh-Tubulin-qRT-PCR-R |

Table S1. Primers used in this study.
